# Diagnostic accuracy of artificial intelligence models for temporomandibular joint anomalies on MRI: a systematic review and meta-analysis

**DOI:** 10.1186/s12938-026-01525-6

**Published:** 2026-01-31

**Authors:** Abhimanyu Pradhan, Aakash Panda, Rajagopal Kadavigere, Neil Abraham Barnes, Suresh Sukumar, Ashwin Prabhu, Dilip Shettigar, Winniecia Dkhar

**Affiliations:** 1 Department of Medical Imaging Technology, Manipal College of Health Professions, Manipal Academy of Higher Education, Manipal, 576104 Karnataka India; 2 Department of Radio Diagnosis and Imaging, Kasturba Medical College, Manipal Academy of Higher Education, Manipal, 576104 Karnataka India

**Keywords:** Artificial intelligence, Magnetic resonance imaging, Temporomandibular joint anomalies, Diagnostic accuracy

## Abstract

**Background:**

Artificial intelligence (AI) techniques are increasingly applied to magnetic resonance imaging (MRI) for detecting temporomandibular joint (TMJ) anomalies; however, their overall diagnostic accuracy and generalizability remain uncertain.

**Objectives:**

To systematically review and meta-analyse the diagnostic performance of AI models for TMJ anomaly detection on MRI and to identify factors influencing model performance.

**Methods:**

A comprehensive search of PubMed, Scopus, Embase, and Web of Science was conducted for studies published between January 2015 and September 2025. Two reviewers independently screened and extracted data. Eligible studies developed and tested AI, machine learning, or deep learning models on human TMJ MRI and reported quantitative performance metrics. Risk of bias was assessed using the QUADAS-2 tool. Pooled sensitivity and specificity were estimated using a bivariate random-effects model, while pooled accuracy was derived using logit transformation. Heterogeneity (*I*^2^) was explored through subgroup analyses by model architecture and validation strategy.

**Results:**

Fourteen studies were included in the systematic review, of which six met the criteria for meta-analysis. Across these six studies, 18 models were analyzed for accuracy, 29 for sensitivity, and 24 for specificity. The pooled diagnostic accuracy was 0.487 (95% CI 0.403–0.571), with pooled sensitivity and specificity of 0.399 (95% CI 0.348–0.450) and 0.399 (95% CI 0.343–0.456), respectively, all showing substantial heterogeneity (*I*^2^ > 90%). Subgroup analyses indicated that advanced architectures such as ResNet-18, Inception v3, and EfficientNet-b4 achieved higher and more consistent diagnostic performance.

**Conclusions:**

Advanced deep learning architectures such as ResNet-18, Inception v3, and EfficientNet-b4 demonstrated superior diagnostic performance for detecting temporomandibular joint anomalies on MRI. These findings highlight the potential of AI-assisted MRI interpretation to improve diagnostic consistency, efficiency, and early detection of TMJ pathology. However, substantial heterogeneity and limited external validation currently limit clinical translation. Standardized multicenter studies and transparent model validation are essential to ensure reliable integration of AI tools into clinical TMJ imaging workflows.

## Introduction

The temporomandibular joint (TMJ) is a pivotal component of the craniofacial skeleton, facilitating mastication, speech, and other essential oral functions [1]. TMJ disorders, which encompass a spectrum of structural and functional abnormalities, can lead to pain, restricted jaw movement, and substantial impairment in daily activities, ultimately affecting an individual’s quality of life. Accurate and early diagnosis of TMJ anomalies is, therefore, critical for effective management and treatment planning [2, 3].

Magnetic resonance imaging (MRI) has become the gold standard for evaluating TMJ disorders due to its superior soft-tissue contrast, non-invasive nature, and ability to visualize both bony and soft-tissue structures, including the articular disc and joint capsule [4–6]. Despite its advantages, MRI interpretation is often time-consuming, subject to inter-observer variability, and reliant on the expertise of radiologists, which may limit diagnostic consistency in clinical practice [7, 8].

In recent years, artificial intelligence (AI) has demonstrated substantial potential in medical imaging, particularly through machine learning and deep learning algorithms capable of automated feature extraction and pattern recognition [9, 10]. AI models have shown promise in enhancing diagnostic accuracy, reducing interpretation time, and providing objective assessments in various musculoskeletal and craniofacial disorders [11, 12].

Given the clinical importance of the TMJ and the challenges in conventional MRI interpretation, this study aims to systematically evaluate the diagnostic accuracy of AI-based algorithms in detecting TMJ anomalies on MRI. By assessing the performance of these models, the study seeks to provide insights into their potential to improve early detection, standardize diagnosis, and support clinical decision-making in TMJ disorders. To our knowledge, this systematic review and meta-analysis to focus exclusively on MRI-based AI models for detecting TMJ anomalies, providing pooled estimates of diagnostic accuracy, sensitivity, and specificity across different architectures. This work addresses a critical evidence gap and offers a foundation for the future clinical translation of AI-assisted TMJ diagnostics.

## Materials and methods

### Study registration

This systematic review was designed and documented by the PRISMA 2020 (Preferred Reporting Items for Systematic Reviews and Meta-Analyses) guidelines [13]. The review protocol was prospectively registered in PROSPERO (CRD420251123040).

### Literature search

A comprehensive literature search was conducted across four major biomedical and medical imaging databases, Scopus, Embase, PubMed, and Web of Science, to identify relevant studies evaluating the efficiency of AI models in detecting TMJ anomalies on MRI. The search was designed to encompass studies published between January 2015 and September 2025, covering various models and their efficacy and accuracy in detecting TMJ anomalies. The final search was conducted on 1 September 2025. To ensure thoroughness and minimize the likelihood of omitting relevant literature, the search strategy incorporated both Medical Subject Headings (MeSH) and free-text keywords, using Boolean operators to combine the following terms: –Key words Temporomandibular joint OR TM Joint OR TM Joint Disorder AND Magnetic Resonance Imaging or MRI AND Radiomics AND AI OR ML OR DL. All retrieved articles were systematically screened by two independent reviewers (A.A.P., and N.A.B.) based on predefined inclusion and exclusion criteria. Duplicate studies were identified and removed using both automatic and manual screening with the help of Rayyan software (https://rayyan.ai/). In addition, the reference lists of all included studies were manually reviewed to identify any additional eligible studies not captured through the initial database search, ensuring the comprehensiveness and reliability of the review process. A third reviewer (W.D.) resolved any disagreements in the selection process. To ensure clinical relevance and methodological rigour, only studies that used human TMJ MRI data, developed or tested an AI-based model, and reported quantitative diagnostic metrics on an independent test dataset were included. This strict focus explains the limited number of included studies relative to the large number initially retrieved.

### Selection of studies

#### Inclusion criteria

Studies were eligible if they were original, peer-reviewed research involving patients with temporomandibular joint (TMJ) disorders or mandibular canal abnormalities assessed using magnetic resonance imaging (MRI). Included studies developed, trained, and tested artificial intelligence (machine learning or deep learning) models on MRI data for detection, classification, segmentation, treatment-response evaluation, or prognosis of TMJ abnormalities. Eligibility required model evaluation on an independent test dataset and reporting of objective quantitative performance metrics (e.g., accuracy, AUC, sensitivity, specificity, F1-score, Dice coefficient, or related indices). Both retrospective and prospective observational or technical validation studies using human MRI data were considered.

#### Exclusion criteria

Studies were excluded if they only reported validation results without testing the model on an independent dataset, or if they relied solely on internal cross-validation techniques without external testing. Articles were excluded if they did not focus specifically on temporomandibular joint (TMJ) abnormalities or mandibular canal–related pathology, or if their primary emphasis was on biomechanical modeling, finite-element analysis, or computer-based simulations unrelated to clinical MRI data. Studies that failed to report essential quantitative performance metrics (e.g., accuracy, AUC, sensitivity, specificity, F1-score, Dice coefficient, or confusion matrix outcomes) were not considered. In addition, studies that utilized only qualitative or semi-quantitative assessments without employing AI- or ML-based quantitative analysis were excluded. Non-original research, such as reviews, conference abstracts, editorials, commentaries, and letters to the editor, was also excluded. Furthermore, studies were excluded if they (a) used imaging modalities other than MRI as the primary input (e.g., CT, CBCT, or panoramic radiography only), (b) lacked an independent test set, (c) reported only descriptive or non-reproducible outcomes, or (d) were based solely on animal, cadaveric, or in-silico phantom data without human clinical MRI datasets. The entire study selection process followed PRISMA guidelines to ensure transparency and reproducibility.

### Data extraction for systematic review

A standardized data extraction form was developed and independently applied by four reviewers (AP, W.D., D.S., and S.S.) to systematically collect relevant information from each included study. The extracted data covered study identifiers (author, year, country), design, sample size, and mean age; imaging parameters such as MRI scanner model, coil type, sequences, acquisition settings (TR, TE, NEX, slice thickness, scan time), and patient positioning; and artificial intelligence–related details including model architecture, sub-model type, dataset distribution (training, validation, and testing), and cross-validation strategies. Performance outcomes were comprehensively documented, encompassing classification and segmentation metrics such as accuracy, AUC, sensitivity, specificity, precision, recall, F1 score, Dice coefficient, positive predictive value (PPV), Hausdorff distance, root mean squared distance, and centroid distance. To ensure clarity and facilitate comparative analysis, the data were synthesized into six structured tables: Table 1 summarizes study and demographic characteristics; Table 2 presents overall model performance metrics; Table 3 reports anatomical region–based outcomes; Table 4 contrasts internal versus external testing results; Table 5 highlights model performance across pathological TMJ conditions; and Table 6 details the effect of layer freezing on YOLO model performance.

**Table 1:** Study characteristics

Sl No	Author, year, Country	Sample size	Training set	Testing set	Mean age	Cross validation	Machine name	Coil name	Sequence	TR(ms)	TE(ms)	NEX	Slice Thickness	Scan Time
1	Ha et al., 2025, South Korea	178	160	18	NM	NM	3.0 T GE Signa	Medium flex-coil	T2 and PDW Sag	2000–3000	50–80	NM	2.5 mm	2:14
2	Azma et al., 2025, Canada	235	135	100	18	NM	A1.5 T & 3 T Siemens	NM	PDW Sag	1800	11	NM	NM	NM
3	Lee et al., 2024, South Korea	NM	NM	NM	NM	fivefold cross	1.5 T GE	21-Channel Head Coil	3D-PDW ZTE	NM	NM	1–2	1 mm	5 min
4	Nozawa et al., Japan, 2024	118	NM	NM	51.3 ± 18.0 42.7 ± 19.9	NM	1.5t GE	Surface Coil	PDW	2000–3000	30	NM	3-4 mm	NM
5	Lee et al., South Korea, 2024	1474	NM	NM	37.19 ± 18.64	NM	3 T GE	Surface Coil	T2 and PDW Sag	2560	82	NM	3 mm	NM
6	Li et al., 2024 China	788	501	167	NM	NM	3 T Siemens	NM	T2W	2000–4000	76–79	NM	3-5 mm	NM
7	Yoon et al., South Korea, 2024	542	383	169	NNM	NM	NM	NM	NM	NM	NM	NM	NM	NM
8	Yoshimi et al., 2024 Japan	49	NM	NM	NM	NM	3 T GE	NM	T2 and PDW Sag	3000	8	1	3 mm	NM
9	Su et al., 2024, Taiwan	194	243	131	NM	fivefold cross	NM	NM	NM	NM	NM	NM	NM	NM
10	Min et al., South Korea, 2024	204	NM	NM	NM	fivefold cross	1.5 T Siemens	NM	T1, T2 and PDW	2250	39	NM	3 mm	NM
11	Yoon et al., 2023, South Korea	502	397	331	Nm	NM	1.5 T Siemens and 3 T Philips	3-inch Surface Coil	NM	400–450	15–20	NM	3 mm	NM
12	Li et al., 2022, China	140	NM	NM	NM	NM	1.5 T and 3 T Siemens	NM	PDW	1800	11	NM	2-3 mm	NM
13	Lee et al., 2022, South Korea	1260	NM	NM	37.33 ± 18.83	NM	3 T GE	Surface Coil	T1, and T2 W	650–2650	14–82	NM	NM	NM
14	Kim et al., 2021, South Korea	289	NM	NM	NM	NM	3 T Philips	3 inch Surface coil	T1, and T2 W	450–2900	20–90	NM	3 mm	NM

NM, Not Mentioned; TR, Repetition Time; TE, Echo Time; NEX, Number of Excitations; PDW, Proton Density Weighted; T1W, T1-weighted; T2W, T2-weighted

**Table 2:** Overall diagnostic performance of AI models for detecting temporomandibular joint (TMJ) anomalies on MRI

Sl no	Author name	Position	Model name	Precision	Recall	Accuracy	F1 Score	AUC	CI 95%	Sensitivity	Specificity	Dice Coefficient	Positive predictive value	Hausdorff distance (mm)
1	Ha et al. (2025)	Open & Close mouth	TransUNet-SegGAN	93.90%	89.97%	NM	NM	NM	NM	NM	NM	78.93%	NM	0.8 mm
2	Lee et al. (2024)	NM	NM	NM	NM	NM	NM	NM	NM	NM	NM	NM	NM	NM
3	Min et al.(2024)	Open & Close mouth	U-Net	NM	NM	NM	NM	NM	NM	0.80 ± 0.13	NM	0.82 ± 0.10	0.87 ± 0.10	8.25 ± 10.31
			U-net + +	NM	NM	NM	NM	NM	NM	0.80 ± 0.12	NM	0.82 ± 0.09	0.85 ± 0.10	8.00 ± 9.32
			U-net 3 +	NM	NM	NM	NM	NM	NM	0.78 ± 0.13	NM	0.81 ± 0.10	0.86 ± 0.10	8.87 ± 12.25
			DeepLabV3 +	NM	NM	NM	NM	NM	NM	0.84 ± 0.09	NM	0.84 ± 0.07	0.85 ± 0.09	6.47 ± 2.22
			SegNet	NM	NM	NM	NM	NM	NM	0.81 ± 0.10	NM	0.83 ± 0.07	0.85 ± 0.10	6.91 ± 7.16
4	Nozawa et al. (2024)	NM	ResNet 18 FM	NM	NM	0.88	NM	0.93	NM	0.87	0.88	NM	NM	NM
			ResNet 18 SM	NM	NM	0.85	NM	0.92	NM	0.86	0.84	NM	NM	NM
			ResNet 18 TM	NM	NM	0.86	NM	0.91	NM	0.83	0.89	NM	NM	NM
			EfficientNet b4 FM	NM	NM	0.81	NM	0.89	NM	0/79	0.83	NM	NM	NM
			EfficientNet b4 SM	NM	NM	0.82	NM	0.9	NM	0.85	0.8	NM	NM	NM
			EfficientNet b4 TM	NM	NM	0.85	NM	0.89	NM	0.88	0.81	NM	NM	NM
			Inception v3 FM	NM	NM	0.86	NM	0.91	NM	0.86	0.85	NM	NM	NM
			Inception v3 SM	NM	NM	0.84	NM	0.9	NM	0.87	0.81	NM	NM	NM
			Inception v3 TM	NM	NM	0.82	NM	0.85	NM	0.73	0.9	NM	NM	NM
			GoogLeNet FM	NM	NM	0.79	NM	0.84	NM	0.84	0.74	NM	NM	NM
			GoogLeNet SM	NM	NM	0.79	NM	0.84	NM	0.74	0.83	NM	NM	NM
			GoogLeNet TM	NM	NM	0.79	NM	0.84	NM	0.85	0.72	NM	NM	NM
5	Lee et al. (2024)	NM	CNN (Fine-tuning PDW)	NM	NM	NM	NM	NM	NM	57.44%	87.25%	NM	77.77%	NM
			CNN (Fine-tuning T2W	NM	NM	NM	NM	NM	NM	57.94%	84.86%	NM	74.83%	NM
			CNN (Ensemble)	NM	NM	NM	NM	NM	NM	52.87%	85.25%	NM	73.57%	NM
6	Li et al. (2024)	Open Mouth	ResNet 152 LR 200 × 200	NM	NM	0.671	NM	0.711	0.625–0.798	0.63	0.76	NM	NM	NM
			ResNet 152 LR 400 × 400	NM	NM	0.695	NM	0.743	0.653–0.834	0.68	0.73	NM	NM	NM
			ResNet 152 NB 200 × 200	NM	NM	0.65	NM	0.707	0.622–0.793	0.59	0.80	NM	NM	NM
			ResNet 152 NB 400 × 400	NM	NM	0.71	NM	0.75	0.666–0.842	0.69	0.76	NM	NM	NM
7	Kim et al. (2021)	NM	MLP	NM	NM	NM	NM	NM	NM	85.20%	84.80%	NM	NM	NM
			Random Forest	NM	NM	NM	NM	NM	NM	96.30%	75.80%	NM	NM	NM
			Disc Shape Alone	NM	NM	NM	NM	NM	NM	80.80%	63.00%	NM	NM	NM
8	Lee et al. (2022)	NM	CNN (Fine Tuning)	NM	NM	0.76	NM	NM	NM	0.65	0.09	NM	NM	NM
			CNN (Ensemble)	NM	NM	0.81	NM	NM	NM	0.82	0.84	NM	NM	NM

NM, Not Mentioned; AUC, Area under the ROC curve; CI, Confidence Interval; PDW, Proton Density Weighted; T2W, T2-weighted; LR, Logistic Regression; NB, Naïve Bayes; MLP, Multilayer Perceptron; PPV, Positive Predictive Value; HD, Hausdorff Distance

**Table 3:** Anatomical region–wise diagnostic performance of AI models in detecting temporomandibular joint (TMJ) substructures

Sl. No	Author name	Position	Evaluation	Region	Model Name	Sensitivity	Specificity	Dice coefficient	Hausdorff distance (mm)	Root squared distance(mm)	Centriod distance(mm)
9	Azma et al. (2025)	Closed Mouth	2D Evaluation	Disc	UNet + +	NM	NM	0.673 ± 0.183	2.181 ± 2.019	0.074 ± 0.101	1.168 ± 1.192
				Condyle	UNet + +	NM	NM	0.915 ± 0.089	0.854 ± 0.569	0.037 ± 0.028	NM
				Eminence	UNet + +	NM	NM	NM	0.966 ± 0.573	0.022 ± 0.012	NM
			3D Evaluation	Disc	UNet + +	NM	NM	0.677 ± 0.1	2.270 ± 1.505	0.016 ± 0.012	1.233 ± 0.847
				Condyle	UNet + +	NM	NM	0.914 ± 0.0411	2.687 ± 8.794	0.013 ± 0.037	NM
				Eminence	UNet + +	NM	NM	NM	2.842 ± 2.304	0.011 ± 0	NM
10	Li et al. (2022)	NM	2D Evaluation	Disc	nnU-Net (3D)	0.70	1	0.70	2.4	0.07	1.13
					Unet + + (2D)	0.65	1	0.70	2.4	0.06	1.09
				Condyle	mu-Net (3D)	0.94	1	0.94	0.64	0.02	NM
					Unet + + (2D)	0.92	1	0.93	0.72	0.03	NM
				Eminence	mu-Net (3D)	NM	NM	NM	0.83	0.02	NM
					Unet + + (2D)	NM	NM	NM	0.88	0.02	NM
			3D Evaluation	Disc	mu-Net (3D)	0.69	1	0.7	2.4	0.01	1.12
					Unet + + (2D)	0.62	1	0.68	2.69	0.02	1.07
				Condyle	mu-Net (3D)	0.94	1	0.93	0.83	0.006	NM
					Unet + + (2D)	0.90	1	0.92	0.93	0.006	NM
				Eminence	mu-Net (3D)	NM	NM	NM	2.12	0.013	NM
					Unet + + (2D)	NM	NM	NM	2.66	0.0014	NM

NM, Not Mentioned; 2D, Two-Dimensional; 3D, Three-Dimensional; Dice, Dice Similarity Coefficient; HD, Hausdorff Distance; RSD, Root Squared Distance; mm, millimeters

**Table 4:** Comparison of internal and external testing performance of AI models for temporomandibular joint (TMJ) anomaly detection

**Sl no**	**Author name**	**Internal Testing**							**External Testing**						
		**Region**	**Model Name**	**AUC**	**Cl 95%**	**Sensitivity**	**Specificity**	**Dice Coefficient**	**Region**	**Model Name**	**AUC**	**Cl 95%**	**Sensitivity**	**Specificity**	**Dice Coefficient**
11	Yoon et al. (2024)	Temporal Bone	DeepLab V-3plus	NM	NM	0.89	0.92	0.85	Temporal Bone	DeepLab V-3plus	NM	NM	0.73	0.822	0.69
			U PerNet-Swin	NM	NM	0.897	0.91	0.85		U PerNet-Swin	NM	NM	0.76	0.84	0.72
			U PerNet-resnet101	NM	NM	0.882	0.90	0.84		U PerNet-resnet101	NM	NM	0.73	0.82	0.69
			OCRNet	NM	NM	0.88	0.90	0.85		OCRNet	NM	NM	0.73	0.82	0.69
			SegFormer	NM	NM	0.91	0.92	0.86		SegFormer	NM	NM	0.77	0.83	0.73
			Ensemble method	NM	NM	0.91	0.92	0.86		Ensemble method	NM	NM	0.76	0.84	0.72
		Disc	DeepLab V-3plus	NM	NM	0.75	0.95	0.71	Disc	DeepLab V-3plus	NM	NM	0.63	0.83	0.60
			U PerNet-Swin	NM	NM	0.75	0.95	0.71		U PerNet-Swin	NM	NM	0.61	0.83	0.56
			U PerNet-resnet101	NM	NM	0.76	0.95	0.72		U PerNet-resnet101	NM	NM	0.62	0.83	0.58
			OCRNet	NM	NM	0.75	0.95	0.71		OCRNet	NM	NM	0.63	0.83	0.60
			SegFormer	NM	NM	0.74	0.95	0.69		SegFormer	NM	NM	0.61	0.83	0.56
			Ensemble method	NM	NM	0.77	0.96	0.73		Ensemble method	NM	NM	0.63	0.83	0.60
		Condyles	DeepLab V-3plus	NM	NM	0.92	0.94	0.89	Condyles	DeepLab V-3plus	NM	NM	0.80	0.83	0.78
			U PerNet-Swin	NM	NM	0.92	0.93	0.89		U PerNet-Swin	NM	NM	0.81	0.84	0.79
			U PerNet-resnet101	NM	NM	0.91	0.93	0.88		U PerNet-resnet101	NM	NM	0.79	0.83	0.77
			OCRNet	NM	NM	0.91	0.93	0.88		OCRNet	NM	NM	0.79	0.83	0.77
			SegFormer	NM	NM	0.93	0.94	0.91		SegFormer	NM	NM	0.81	0.84	0.78
			Ensemble method	NM	NM	0.92	0.94	0.91		Ensemble method	NM	NM	0.81	0.84	0.8
		Background	DeepLab V-3plus	NM	NM	0.89	0.86	0.95	Background	DeepLab V-3plus	NM	NM	0.822	0.80	0.86
			U PerNet-Swin	NM	NM	0.89	0.86	0.94		U PerNet-Swin	NM	NM	0.82	0.80	0.87
			U PerNet-resnet101	NM	NM	0.89	0.86	0.94		U PerNet-resnet101	NM	NM	0.82	0.80	0.86
			OCRNet	NM	NM	0.89	0.86	0.94		OCRNet	NM	NM	0.81	0.80	0.86
			SegFormer	NM	NM	0.89	0.86	0.94		SegFormer	NM	NM	0.82	0.80	0.86
			Ensemble method	NM	NM	0.89	0.86	0.95		Ensemble method	NM	NM	0.82	0.80	0.86
12	Yoon et al. (2023)	NM	Detection Model(0.819)	0.98	0.976,0.997	0.95	0.92	NM	NM	Detection Model(0.819)	0.956	0.926,0.979)	0.91	0.9	NM
			ADcR	0.93	0.909,0.961	0.88	0.81	NM		ADCR	0.93	(0.902,0.966)	0.91	0.83	NM
			ADsR	0.91	(0.977,1.00)	0.96	0.94	NM		ADsR	0.98	0.974,0.99	0.94	0.94	NM

NM, Not Mentioned; AUC, Area under the ROC curve; CI, Confidence Interval; ADcR, Anterior Disc reduction; ADsR, Anterior Disc shift reduction

**Table 5:** Performance of AI models in detecting temporomandibular joint (TMJ) pathological conditions under different evaluation settings

Sl no	Author Name	Position	Evaluation	Pathological Condition	Model	Sensitivity	Specificity	Dice Coefficient	PPV	Hausdorff distance (mm)	Root Squared Distance(mm)	Centriod Distance(mm)	Disk Classification Accuracy
9	Amza et al. (2025)	Closed Mouth	2D Evalauation	Non-Dislocated	UNet + +	NM	NM	0.677 ± 0.183	NM	2.107 ± 2.047	0.0715 ± 0.108	1.172 ± 1.247	0.927
				Dislocated	UNet + +	NM	NM	0.654 ± 0.181	NM	2.504 ± 1.879	0.085 ± 0.065	1.148 ± 0.92	0.89
			3D Evaluation	Non-Dislocated	UNet + +	NM	NM	0.690 ± 0.092	NM	2.040 ± 1.226	0.014 ± 0	1.127 ± 0.728	NM
				Dislocated	UNet + +	NM	NM	0.625 ± 0.111	NM	3.189 ± 2.064	0.027 ± 0.021	1.657 ± 1.119	NM
13	Yoshimi et al. (2024)	Closed Mouth	Experiment 1 Original Image	Normal	ED-CNN	0.77 ± 0.21	NM	0.62 ± 0.20	0.56 ± 0.24	NM	NM	NM	NM
			CLAHE			0.80 ± 0.12	NM	0.66 ± 0.14	0.59 ± 0.19	NM	NM	NM	NM
			Original Image	Displaced		0.71 ± 0.34	NM	0.65 ± 0.32	0.63 ± 0.33	NM	NM	NM	NM
			CLAHE			0.77 ± 0.25	NM	0.71 ± 0.24	0.68 ± 0.26	NM	NM	NM	NM
			Original Image	Both		0.74 ± 0.28	NM	0.64 ± 0.24	0.60 ± 0.25	NM	NM	NM	NM
			CLAHE			0.74 ± 0.23	NM	0.67 ± 0.21	0.64 ± 0.24	NM	NM	NM	NM
		Open Mouth	Original Image	Normal		0.70 ± 0.29	NM	0.66 ± 0.25	0.66 ± 0.27	NM	NM	NM	NM
			CLAHE			0.79 ± 0.19	NM	0.73 ± 0.17	0.71 ± 0.20	NM	NM	NM	NM
			Original Image	Displaced		0.67 ± 0.34	NM	0.65 ± 0.33	0.65 ± 0.34	NM	NM	NM	NM
			CLAHE			0.75 ± 0.27	NM	0.74 ± 0.25	0.74 ± 0.26	NM	NM	NM	NM
			Original Image	Both		0.80 ± 0.23	NM	0.73 ± 0.19	0.72 ± 0.19	NM	NM	NM	NM
			CLAHE			0.78 ± 0.23	NM	0.75 ± 0.21	0.74 ± 0.22	NM	NM	NM	NM
		Open and Closed	Original Image	Normal		0.73 ± 0.26	NM	0.64 ± 0.23	0.61 ± 0.26	NM	NM	NM	NM
			CLAHE			0.79 ± 0.16	NM	0.70 ± 0.16	0.66 ± 0.21	NM	NM	NM	NM
			Original Image	Displaced		0.69 ± 0.34	NM	0.65 ± 0.32	0.64 ± 0.34	NM	NM	NM	NM
			CLAHE			0.76 ± 0.25	NM	0.73 ± 0.25	0.71 ± 0.26	NM	NM	NM	NM
			Original Image	Both		0.77 ± 0.26	NM	0.69 ± 0.22	0.66 ± 0.22	NM	NM	NM	NM
			CLAHE			0.76 ± 0.23	NM	0.71 ± 0.21	0.69 ± 0.24	NM	NM	NM	NM
		Closed Mouth	Experiment 2 Original Image	Normal		0.61 ± 0.24	NM	0.61 ± 0.21	0.65 ± 0.24	NM	NM	NM	NM
			CLAHE			0.57 ± 0.32		0.56 ± 0.28	0.63 ± 0.29	NM	NM	NM	NM
			Original Image	Displaced		0.49 ± 0.41	NM	0.50 ± 0.41	0.52 ± 0.43	NM	NM	NM	NM
			CLAHE			0.64 ± 0.39	NM	0.61 ± 0.35	0.62 ± 0.36	NM	NM	NM	NM
			Original Image	Both		0.40 ± 0.38	NM	0.41 ± 0.37	0.46 ± 0.4	NM	NM	NM	NM
			CLAHE			0.56 ± 0.29	NM	0.61 ± 0.35	0.72 ± 0.30	NM	NM	NM	NM
		Open Mouth	Original Image	Normal		0.35 ± 0.41	NM	0.35 ± 0.40	0.36 ± 0.41	NM	NM	NM	NM
			CLAHE			0.68 ± 0.28	NM	0.76 ± 0.26	0.69 ± 0.26	NM	NM	NM	NM
			Original Image	Displaced		0.28 ± 0.36	NM	0.31 ± 0.36	0.37 ± 0.42	NM	NM	NM	NM
			CLAHE			0.59 ± 0.37	NM	0.59 ± 0.34	0.63 ± 0.36	NM	NM	NM	NM
			Original Image	Both		0.27 ± 0.38	NM	0.27 ± 0.37	0.32 ± 0.41	NM	NM	NM	NM
			CLAHE			0.64 ± 0.33	NM	0.64 ± 0.31	0.68 ± 0.33	NM	NM	NM	NM
		Open and Closed	Original Image	Normal		0.48 ± 0.35	NM	0.48 ± 0.34	0.51 ± 0.36	NM	NM	NM	NM
			CLAHE			0.62 ± 0.31	NM	0.63 ± 0.28	0.69 ± 0.28	NM	NM	NM	NM
			Original Image	Displaced		0.38 ± 0.39	NM	0.39 ± 0.39	0.43 ± 0.43	NM	NM	NM	NM
			CLAHE			0.62 ± 0.38	NM	0.60 ± 0.34	0.62 ± 0.36	NM	NM	NM	NM
			Original Image	Both		0.33 ± 0.38	NM	0.34 ± 0.37	0.38 ± 0.41	NM	NM	NM	NM
			CLAHE			0.60 ± 0.31	NM	0.63 ± 0.30	0.70 ± 0.31	NM	NM	NM	NM

NM, Not Mentioned; PPV, Positive Predictive Value; CLAHE, Contrast Limited Adaptive Histogram Equalization

**Table 6:** Accuracy of YOLO model for temporomandibular joint (TMJ) detection under open-mouth condition with varying freezing layers

Sl No.	Author	Position	Freezing layer	Model name	Accuracy	Mean value ± 1 standard deviation
1	Su et al. [28]	Open Mouth	0	YOLO	0.7251	0.6856–0.7647
			1		0.7362	0.6936–0.7788
			3		0.7408	0.6926–0.7890
			4		0.7405	0.6933–0.787
			6		0.7285	0.6796–0.7774
			7		0.7291	0.6991–0.7592
			8		0.7147	0.6787–0.7508
			10		0.7129	0.6800–0.7459
			11		0.6957	0.6605–0.7309
			12		0.6924	0.6594–0.7254
			14		0.6578	0.5990–0.7167
			15		0.6303	0.5890–0.6717
			16		0.5775	0.5265–0.6285
			18		0.4682	0.4472–0.4893

YOLO, You Only Look Once; TMJ, Temporomandibular Joint

### Data extraction for meta-analysis

Meta-analysis was conducted by two independent reviewers (N.A.B. and A.S.P.) to quantitatively evaluate the diagnostic performance of artificial intelligence models applied to MRI in the detection and classification of temporomandibular joint (TMJ) abnormalities. For inclusion in the quantitative synthesis, outcome measures were required to be reported by at least three independent studies. In the present study, the pooled analysis focused on three key performance metrics: accuracy (reported in 3 studies), sensitivity (6 studies), and specificity (5 studies).

When studies reported sensitivity or specificity in the form of mean ± standard deviation, the standard error (SE) was derived using the formula *SE* = *SD/√N* to enable uniform comparison and pooling. Subgroup analyses were performed separately for accuracy, sensitivity, and specificity to evaluate heterogeneity and model-specific performance across studies. This structured approach ensured that diagnostic accuracy was assessed consistently while accounting for variations in reporting formats. All extracted data were standardized and coded before pooling to maintain methodological rigor and facilitate cross-study comparability.

### Quality assessment

The methodological quality and risk of bias of each included study were evaluated using the Quality Assessment of Diagnostic Accuracy Studies-2 (QUADAS-2) [14]. This tool is specifically designed for diagnostic accuracy research and assesses risk of bias across four domains: Patient Selection, Index Test, Reference Standard, and Flow and Timing. Additionally, the first three domains were evaluated for applicability concerns. Each domain was rated as having low risk, high risk, or unclear risk based on the predefined signaling questions. Two reviewers (S.S., R.K.) independently assessed each study, and any disagreements were resolved by consensus or with the input of a third reviewer (A.P.)

### Statistical analysis

All statistical analyses were performed using Jamovi software (Version 2.6; www.jamovi.org). The primary outcomes of interest were the diagnostic performance metrics of AI-based models, including accuracy, sensitivity, and specificity. For each metric, pooled estimates and corresponding 95% confidence intervals (CIs) were calculated using a meta-analysis of proportions.

Heterogeneity across studies was quantified using the I^2^ statistic and Cochran’s Q test. In cases where substantial heterogeneity was observed (I^2^ > 50%), a random-effects model was applied; otherwise, a fixed-effect model was used. Subgroup analyses were conducted based on model architecture and study cohort to explore potential sources of heterogeneity and provide more robust pooled estimates.

All results are presented as pooled effect sizes with 95% CIs, along with measures of heterogeneity to ensure transparency and reliability of the findings.

## Result

A comprehensive literature search was conducted across four major databases: PubMed (*n* = 10,187), Scopus (*n* = 963), Embase (*n* = 63), and Web of Science (*n* = 2241). After removing 185 duplicate records, 13,269 unique articles were subjected to title screening. Of these 13,189 studies were excluded for failing to meet the predefined inclusion criteria, most commonly due to the absence of relevant pathology or a primary focus on non-clinical applications such as biomechanical or computational modeling. 80 articles were selected for full-text retrieval. However, 22 studies could not be accessed due to availability constraints. The remaining 58 articles underwent detailed eligibility assessment, leading to the exclusion of 44 studies owing to issues such as insufficient reporting of model performance metrics, inclusion of only validation datasets, use of other imaging modalities, or incomplete model evaluations.

**Fig. 1:**
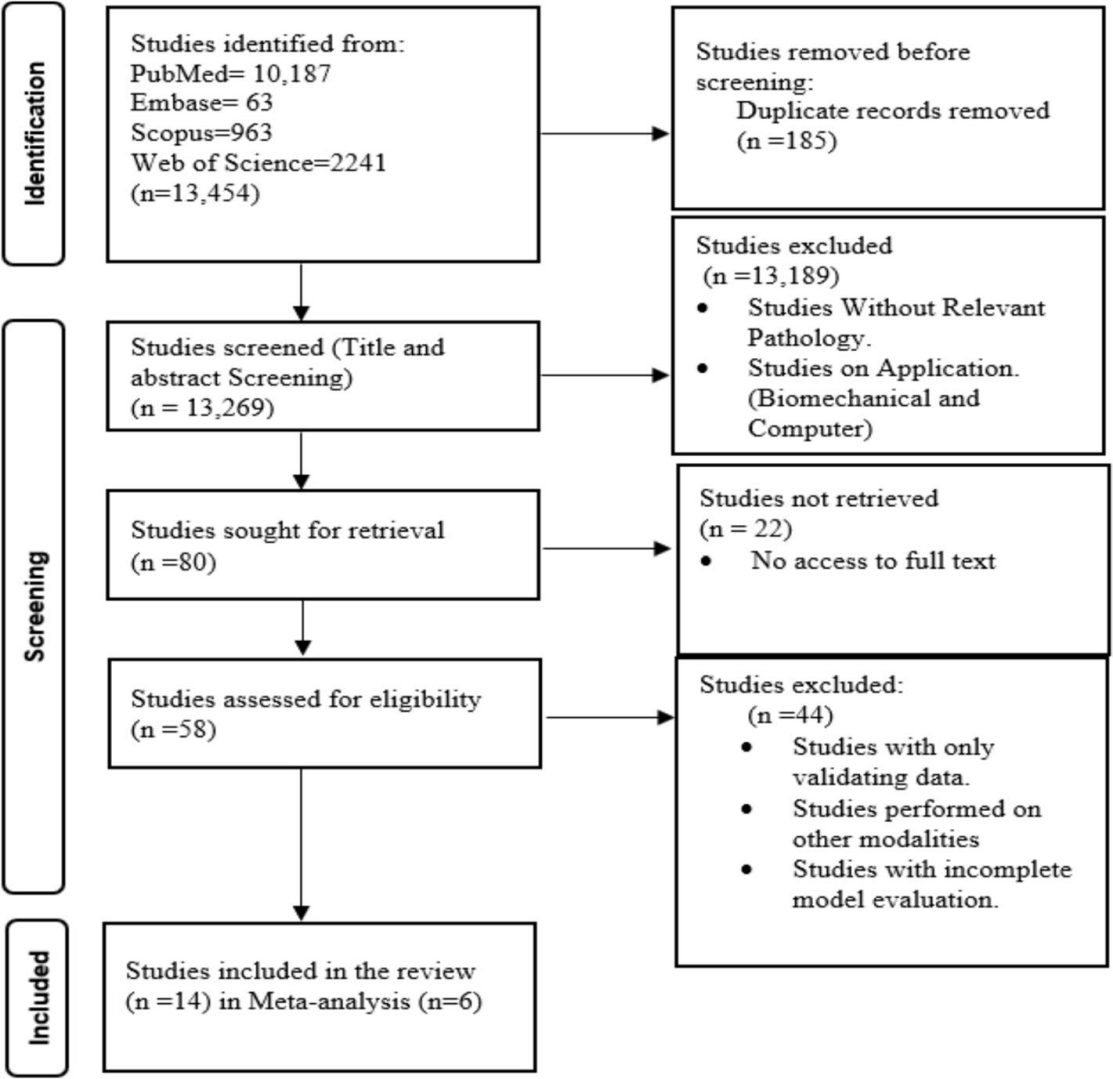
PRISMA chart

At the end, a total of 14 studies met all the inclusion criteria for systematic review, which were incorporated into the final systematic review. The study selection process is outlined in the PRISMA flow diagram (Fig. 1).

### Study characteristics

A total of 14 studies were included in this systematic review, originating from South Korea [15–22], Japan [23, 24], China [25], Canada [26, 27], and Taiwan [28]. All Studies employed retrospective designs and reported obtaining appropriate ethical approval. Each study aimed to develop machine learning (ML) or deep learning (DL) models for detecting TMJ anomalies. The sample sizes across these studies ranged substantially, from to participants, with reported mean/median ages typically falling between and years. All studies utilized Magnetic Resonance Imaging (MRI) data. The MRI acquisition protocol showed significant technical heterogeneity, predominantly employing Proton Density (PD)-weighted and -weighted sagittal sequences. Imaging parameters varied widely, with Repetition Time () ranging from approximately to and Echo Time ranging from to. Furthermore, the imaging was performed using various coils (such as a Channel Head Coil or Medium Flex Coil) with slice thicknesses generally set between 2.5 mm and 5 mm. Model architectures varied widely, encompassing advanced networks such as TransUNet-SegGAN, ResNet 152, UNet +  + , nnU-Net, DeepLab V3 + , and various U PerNet models. Segmentation accuracy was predominantly measured using the Dice Coefficient (DC) and Hausdorff Distance (HD). For organized discussion of results, without implying sub-analysis, the studies were grouped based on the type of performance evaluation reported: (1) Overall Performance (as detailed in the Data Extraction.xlsx—Overall Performance sheet), which reported fundamental metrics like Dice Coefficient, Sensitivity, and Specificity; (2) Anatomical Region Wise Performance (as detailed in the Data Extraction.xlsx—Anat Region Wise Performance sheet), which provided segregated metrics for the Disk, Condyle, and Eminence; (3) Internal and External Testing (as detailed in the Data Extraction.xlsx—Internal Test and External Test sheet), which evaluated model generalizability using separate, independent external datasets; and (4) Disease Stage Specific (as detailed in the Data Extraction.xlsx—Diesease Stage Specific sheet), which focused on classification accuracy under specific conditions like non-dislocated versus dislocated TMJ or across different disease stages.. These organizational groupings are summarized in Tables 1–6, which detail the study designs, sample characteristics, model types, and diagnostic performance metrics.

### Quality assessment

**Fig. 2:**
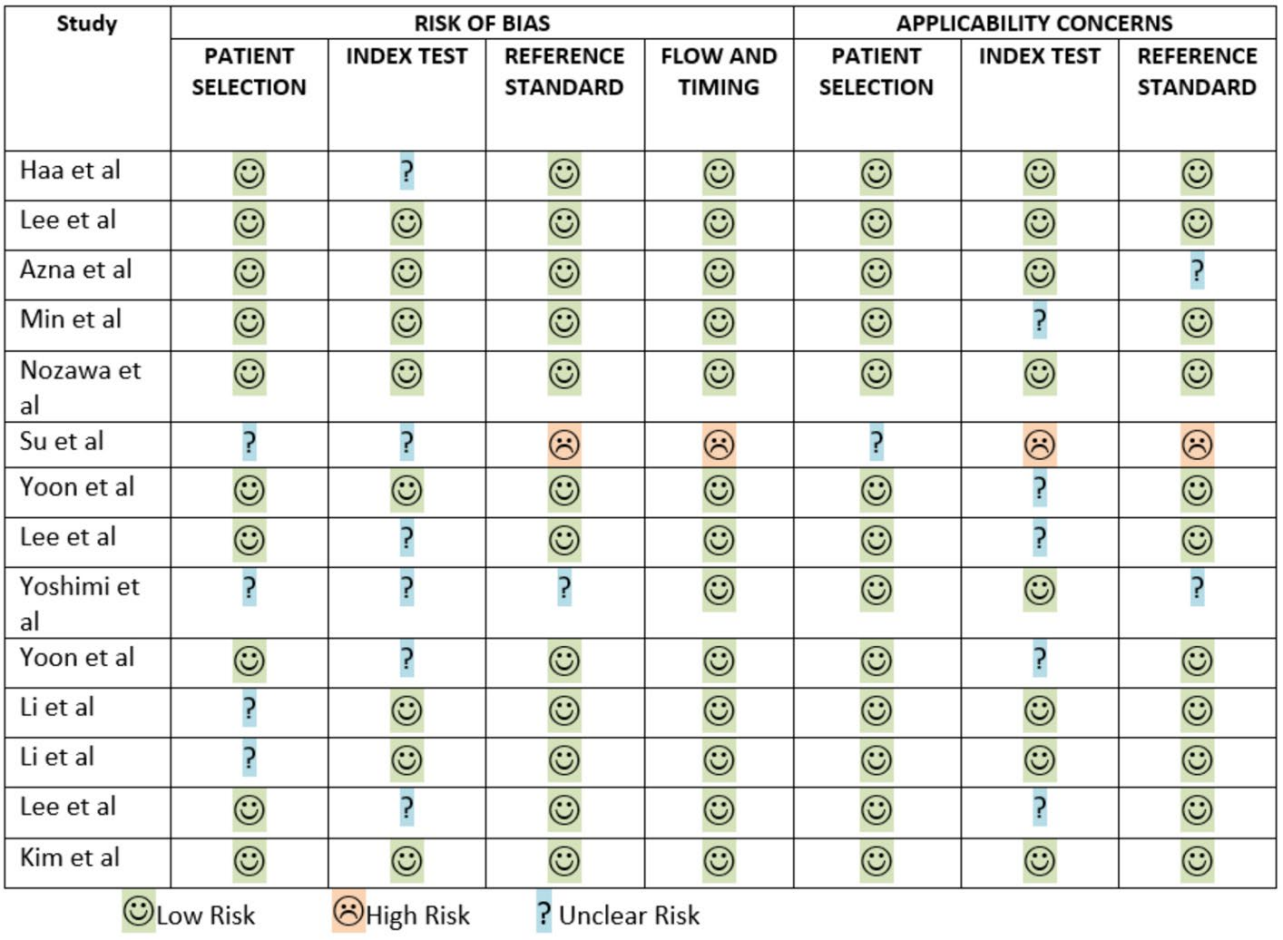
Quality assessment of the articles included in the systematic review and meta-analysis. Haa et al. [15], Lee et al. [18], Azma et al. [26], Min et al., [17], Nozawa et al. [23], Su et al. [28], Yoon et al. [21], Lee et al.[18], Yoshimi et al. [24], Yoon et al. [22], Li et al. [25], Li et al. [27], Lee et al. [20], Kim et al. [19]

The methodological quality and applicability of the fourteen included studies were evaluated using the QUADAS-2 tool across the domains of Patient Selection, Index Test, Reference Standard, and Flow and Timing. Among these, Kim et al. [19], Lee et al. [16], and Nozawa et al. [23] exhibited the most robust methodology, with all domains rated as Low Risk for both bias and applicability. Haa et al. [15] and Li et al. [25] showed an Unclear Risk of bias only in Patient Selection, while Lee et al. [20], and Li et al. [27] demonstrated Unclear applicability concerns in the Reference Standard domain. Min et al. [17], Yoon et al. [22], and Yoon et al. [21] presented Unclear applicability in Patient Selection. In contrast, Azna et al. [26] showed Unclear Risk of bias in the Reference Standard and Unclear applicability in Patient Selection. Lee et al. [18] demonstrated Unclear Risk of bias in both Index Test and Flow and Timing, and Unclear applicability in the Reference Standard. Yoshimi et al. [24] identified multiple concerns, including Unclear Risk of bias in Patient Selection, Index Test, and Reference Standard, as well as unclear applicability in the Reference Standard. The study by Su et al. [28] had the highest risk, with Unclear Risk of bias in Patient Selection, Index Test, and Flow and Timing, and Unclear applicability in Patient Selection. Overall, the most common methodological limitation across studies was Unclear Risk of bias and applicability concern in the Patient Selection domain, indicating potential heterogeneity in recruitment processes and limited transparency in reporting participant characteristics. The assessment is schematically represented in Fig. 2.

### Meta-analysis

A meta-analysis was conducted to quantitatively evaluate the predictive performance of machine learning (ML) and deep learning (DL) models for temporomandibular joint anomaly detection on MRI. Because not all studies reported the same diagnostic metrics, the number of datasets included in each pooled analysis varied. Data from 18 model datasets were pooled to assess overall accuracy, yielding a value of 0.487 (95% CI 0.403–0.571) under a random-effects model, indicating moderate diagnostic performance. Similarly, 29 datasets were analyzed to estimate pooled sensitivity, which was 0.399 (95% CI 0.348–0.450), and 24 datasets were used to calculate pooled specificity, which was 0.399 (95% CI 0.343–0.456). Substantial heterogeneity was observed across all analyses (I^2^ > 90%), likely reflecting differences in dataset characteristics, model architectures, imaging parameters, and training strategies. To address this variability, subgroup analyses were performed to obtain more precise estimates within homogeneous model groups and explore potential sources of heterogeneity.

### Overall pooled accuracy

A total of eighteen models derived from four included studies were analyzed. The accuracy of individual models demonstrated considerable variability, ranging from 0.051 (95% CI 0.041–0.064; Lee et al., Fine Tuning) to 0.746 (95% CI 0.660–0.816; Nozawa et al., ResNet-18 FM). Models based on ResNet and Inception architectures consistently achieved higher accuracies (0.69–0.75), whereas ensemble and fine-tuning approaches generally performed less effectively (< 0.10).

The pooled meta-analytic estimate of diagnostic accuracy, calculated using a random-effects model, was 0.487 (95% CI 0.403–0.571) across all included models (*k* = 18). This indicates a moderate overall performance of AI-based approaches in the studies included.

However, heterogeneity across studies was substantial (*I*^2^ = 99.36%; *τ*^2^ = 0.0519; *H*^2^ = 257.9), reflecting marked variability in model architecture, training strategies, and dataset characteristics. The corresponding forest plot visually illustrates this wide distribution of effect sizes, with some models demonstrating comparatively high diagnostic performance while others yielded minimal discriminative capability.

**Fig. 3:**
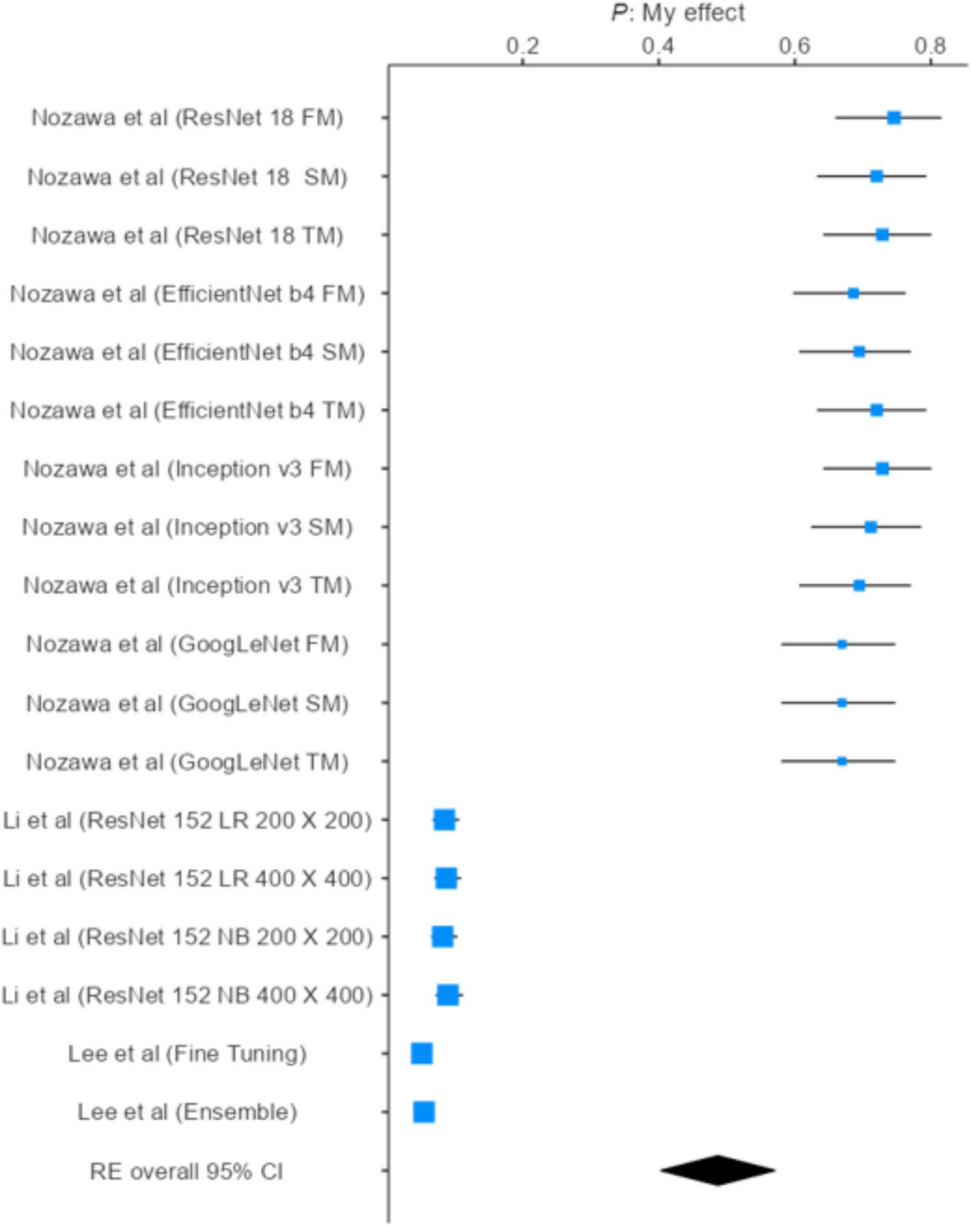
Accuracy estimates of AI-based diagnostic models

Given this level of heterogeneity, the pooled effect should be interpreted with caution. These findings underscore the need for further standardization of model training and validation protocols to enhance the comparability and reliability of diagnostic accuracy estimates across studies [20, 23, 25] (Fig. 3).

### Subgroup analysis of accuracy-based models

#### Subgroup analysis of the ResNet-18 model

**Fig. 4:**
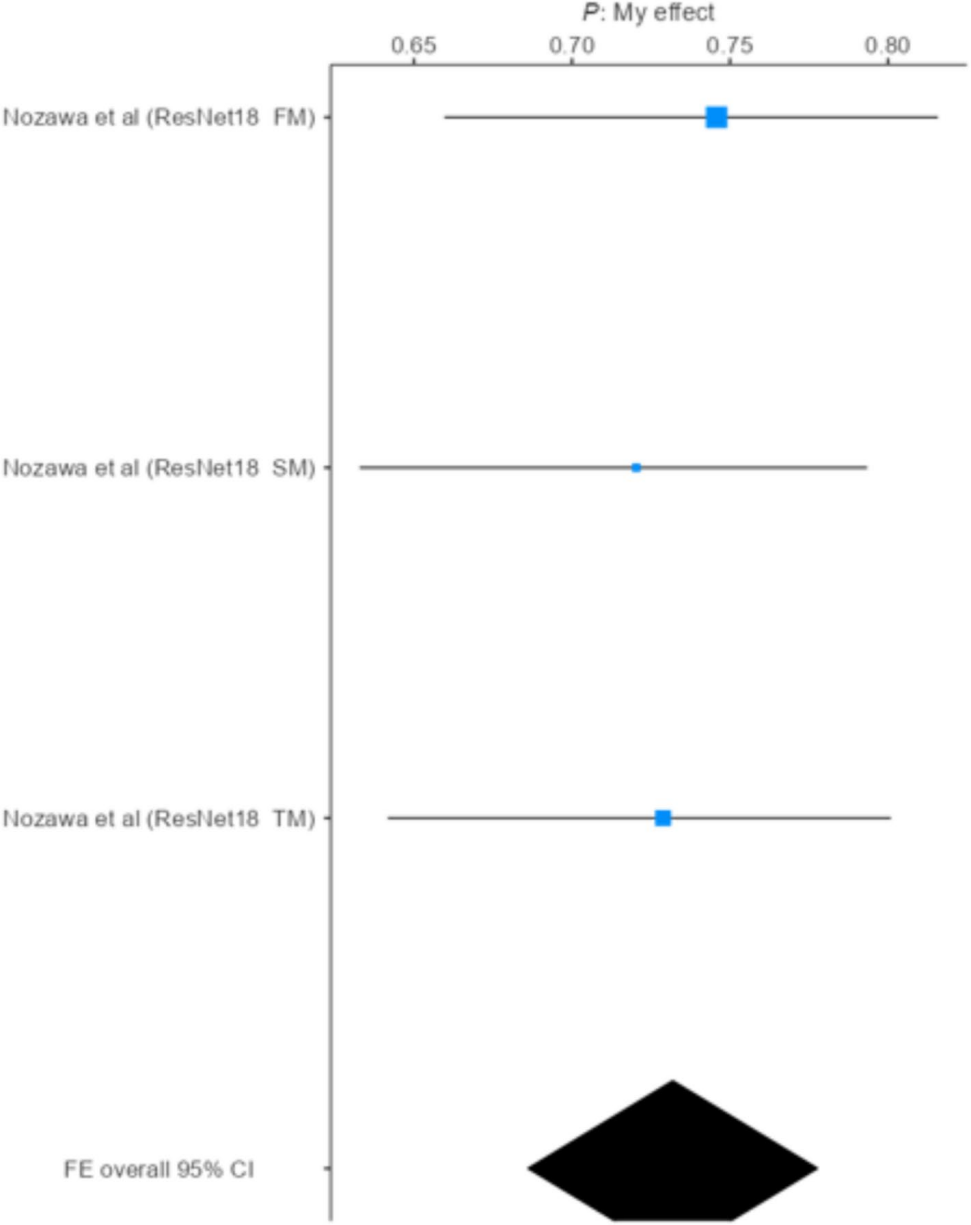
Accuracy estimates of ResNet-18 diagnostic models

Subgroup analyses were conducted to investigate differences in accuracy across various model architectures. For the three datasets included, models based on the ResNet-18 architecture achieved pooled accuracies ranging from 0.720 to 0.746. The meta-analytic pooled effect size for this subgroup was 0.732 (95% CI 0.686–0.778), calculated using a fixed-effect model due to the absence of heterogeneity (*I*^2^ = 0%). This indicates that the accuracy estimates among ResNet-18 models were highly consistent, suggesting stable performance within this architectural subgroup. The lack of variability across studies implies that differences in dataset characteristics or training procedures had minimal impact on model performance within this subgroup [23] (Fig. 4).

#### Subgroup analysis of inception v3 models

Subgroup analysis was conducted for three datasets utilizing Inception v3 architectures. Individual accuracies ranged from 0.695 (95% CI 0.606–0.771) to 0.729 (95% CI 0.642–0.801). The pooled accuracy was 0.712 (95% CI 0.666–0.759), calculated using a fixed-effect model, as heterogeneity was negligible (*I*^2^ = 0%). These findings indicate consistent performance of Inception v3 models across the included studies, suggesting stable predictive capability within this architectural subgroup [23]. (Supplementary Fig. 1).

#### Subgroup analysis of EfficientNet b4 model

Subgroup analysis was performed for three datasets employing EfficientNet b4 architectures. Individual accuracies ranged from 0.686 (95% CI 0.598–0.763) to 0.720 (95% CI 0.633–0.793). The pooled accuracy was 0.701 (95% CI 0.654–0.748), calculated using a fixed-effect model due to negligible heterogeneity (*I*^2^ = 0%). These results indicate that EfficientNet b4 models demonstrated consistent predictive performance across the included studies, suggesting reliable accuracy within this architectural subgroup [23] (Supplementary Fig. 2).

#### Subgroup analysis of GoogLeNet model

Subgroup analysis was conducted for three datasets employing GoogLeNet architectures. All models demonstrated an identical accuracy of 0.669, with 95% confidence intervals ranging from 0.580 to 0.748. The pooled accuracy was 0.669 (95% CI 0.621–0.718), calculated using a fixed-effect model, consistent with negligible heterogeneity (*I*^2^ = 0%). These results indicate highly consistent performance of GoogLeNet models across the included datasets, reflecting stable predictive capability within this architectural subgroup [23] (Supplementary Fig. 3).

#### Subgroup analysis of ResNet 152 model

Subgroup analysis was performed for four datasets employing ResNet 152 architectures with varying image resolutions (200 × 200 and 400 × 400) and learning strategies (LR vs. NB). Individual accuracies ranged from 0.0825 to 0.0901 (95% CI 0.065–0.112). The pooled accuracy was 0.0862 (95% CI 0.0763–0.0961), calculated using a fixed-effect model due to negligible heterogeneity (*I*^2^ = 0%). These results indicate consistently low predictive performance of ResNet 152 models across all datasets, suggesting limited effectiveness within this subgroup [25] (Supplementary Fig. 4).

#### Pooled sensitivity estimate

**Fig. 5:**
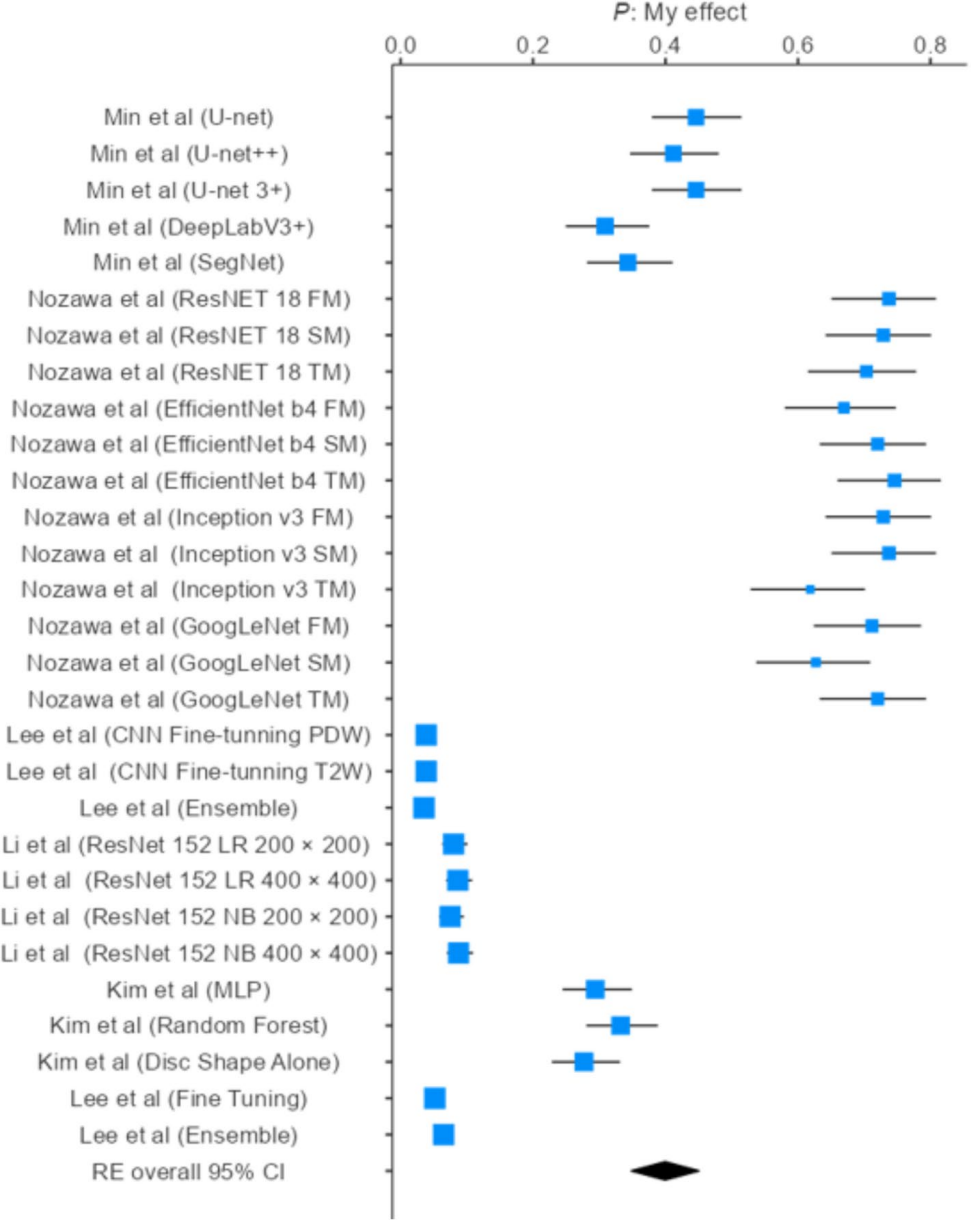
Sensitivity estimates of AI-based diagnostic models

A meta-analysis of 29 datasets across 6 studies were performed to assess the sensitivity of machine learning and deep learning models. Individual sensitivities varied widely, ranging from 0.035 to 0.746, reflecting differences in model architecture, dataset characteristics, and training approaches. The pooled sensitivity was 0.399 (95% CI 0.348–0.450) using a random-effects model. Substantial heterogeneity was observed (*I*^2^ = 99.23%), indicating that variability among studies was considerable. These results highlight moderate overall sensitivity of the included models and underscore the need for subgroup analyses to further explore sources of variability and identify model-specific performance patterns [17–20, 23, 25] (Fig. 5).

### Subgroup analysis of the sensitivity-based models

#### Subgroup analysis of U-net models and other architectures

A subgroup meta-analysis was conducted for three datasets employing U-net-based architectures, including U-net, U-net +  + , and U-net 3 + . Individual sensitivities ranged from 0.412 to 0.446, with 95% confidence intervals of 0.347–0.515. The pooled sensitivity was 0.401 (95% CI 0.353–0.450), calculated using a random-effects model. Moderate heterogeneity was observed among the included studies (*I*^2^ ≈ 71.6%), suggesting that variability in model variants and training approaches influenced performance. These results indicate moderate predictive sensitivity for U-net models in this analysis, highlighting the need for model-specific optimization.

For DeepLabV3 + and SegNet, sensitivities were lower, ranging from 0.309 to 0.343, with individual confidence intervals of 0.250–0.411. The pooled sensitivity for these architectures was 0.326 (95% CI 0.283–0.369), reflecting moderate-to-low predictive performance [17] (Supplementary Fig. 5).

#### Subgroup analysis of GoogLeNet model

A subgroup meta-analysis was conducted for three datasets using GoogLeNet architectures (FM, SM, and TM variants) to evaluate sensitivity. Individual sensitivity values ranged from 0.627 to 0.720, with 95% confidence intervals spanning 0.537–0.793. The pooled sensitivity, calculated using a fixed-effects model, was 0.689 (95% CI 0.641–0.736). Heterogeneity among these studies was low-to-moderate, as reflected by a Diamond Ratio of 1.20, indicating relatively consistent performance across GoogLeNet variants. The corresponding forest plot illustrates individual study sensitivities and the pooled estimate, highlighting that GoogLeNet models achieved moderate-to-high sensitivity in this analysis [23] (Supplementary Fig. 6).

#### Subgroup analysis of EfficientNet b4 model

A subgroup meta-analysis was performed for three datasets using EfficientNet b4 architectures (FM, SM, and TM variants) to evaluate sensitivity. Individual sensitivity values ranged from 0.669 to 0.746, with 95% confidence intervals spanning 0.580–0.816. The pooled sensitivity, calculated using a fixed-effects model, was 0.714 (95% CI 0.667–0.760), reflecting relatively consistent performance across these variants. Heterogeneity was minimal-to-moderate, as indicated by a Diamond Ratio of 1.00, suggesting homogeneity of effect sizes within this subgroup. The forest plot illustrates both individual study estimates and the pooled effect, indicating that EfficientNet b4 models achieved moderate-to-high sensitivity in the analyzed datasets [23] (Supplementary Fig. 7).

#### Subgroup analysis of inception v3 model

A subgroup meta-analysis was conducted for three datasets using Inception v3 architectures (FM, SM, and TM variants) to evaluate sensitivity. Individual sensitivity values ranged from 0.619 to 0.737, with 95% confidence intervals spanning 0.528–0.808. The pooled sensitivity, calculated using a random-effects model, was 0.697 (95% CI 0.624–0.769), indicating moderate-to-high performance. Heterogeneity was moderate (*I*^2^ = 58.1%), suggesting some variability across datasets, potentially attributable to differences in imaging parameters, model training strategies, or dataset composition. The forest plot illustrates both individual study sensitivities and the pooled estimate, highlighting that Inception v3 models achieved consistent sensitivity while accounting for inter-study variability [23] (Supplementary Fig. 8).

#### Subgroup analysis of ResNet 18 model

A subgroup meta-analysis was performed on three datasets employing ResNet 18 variants (FM, SM, and TM) to assess sensitivity. Individual study sensitivities ranged from 0.703 to 0.737, with 95% confidence intervals between 0.615 and 0.808. The pooled sensitivity, estimated using a fixed-effects model, was 0.724 (95% CI 0.677–0.770), reflecting high sensitivity across these datasets. Heterogeneity was minimal, as indicated by a Diamond Ratio of 1.00, supporting the use of a fixed-effects approach. The forest plot demonstrates consistent sensitivity among the ResNet 18 models, suggesting robust predictive performance and low inter-study variability [23] (Supplementary Fig. 9).

#### Subgroup analysis of CNN models

A subgroup meta-analysis was performed on three datasets from Lee et al., which included CNN Fine-Tuning PDW, CNN Fine-Tuning T2W, and an Ensemble model. Individual study sensitivities were consistently low, ranging from 0.0353 to 0.0387 with 95% confidence intervals of 0.0270–0.0499. The pooled sensitivity, estimated using a fixed-effects model, was 0.0375 (95% CI 0.0318–0.0432). Heterogeneity across these studies was minimal, indicated by a Diamond Ratio of 1.00, justifying the use of a fixed-effects model. The forest plot illustrates the uniform low sensitivity across all three models, suggesting limited predictive performance within this subgroup [18] (Supplementary Fig. 10).

#### Subgroup analysis of ResNet 152 model

A subgroup meta-analysis was conducted on four datasets from Li et al., including ResNet 152 LR 200 × 200, LR 400 × 400, NB 200 × 200, and NB 400 × 400 models. Individual study sensitivities ranged from 0.0749 to 0.0876, with 95% confidence intervals between 0.0585 and 0.1095. The pooled sensitivity, estimated using a fixed-effects model, was 0.0819 (95% CI 0.0722–0.0915). Minimal heterogeneity was observed across these datasets, as indicated by a Diamond Ratio of 1.00, supporting the appropriateness of the fixed-effects approach. The forest plot demonstrates uniformly low sensitivity across all four models, reflecting limited predictive performance within this subgroup [25] (Supplementary Fig. 11).

#### Pooled specificity estimate

**Fig. 6:**
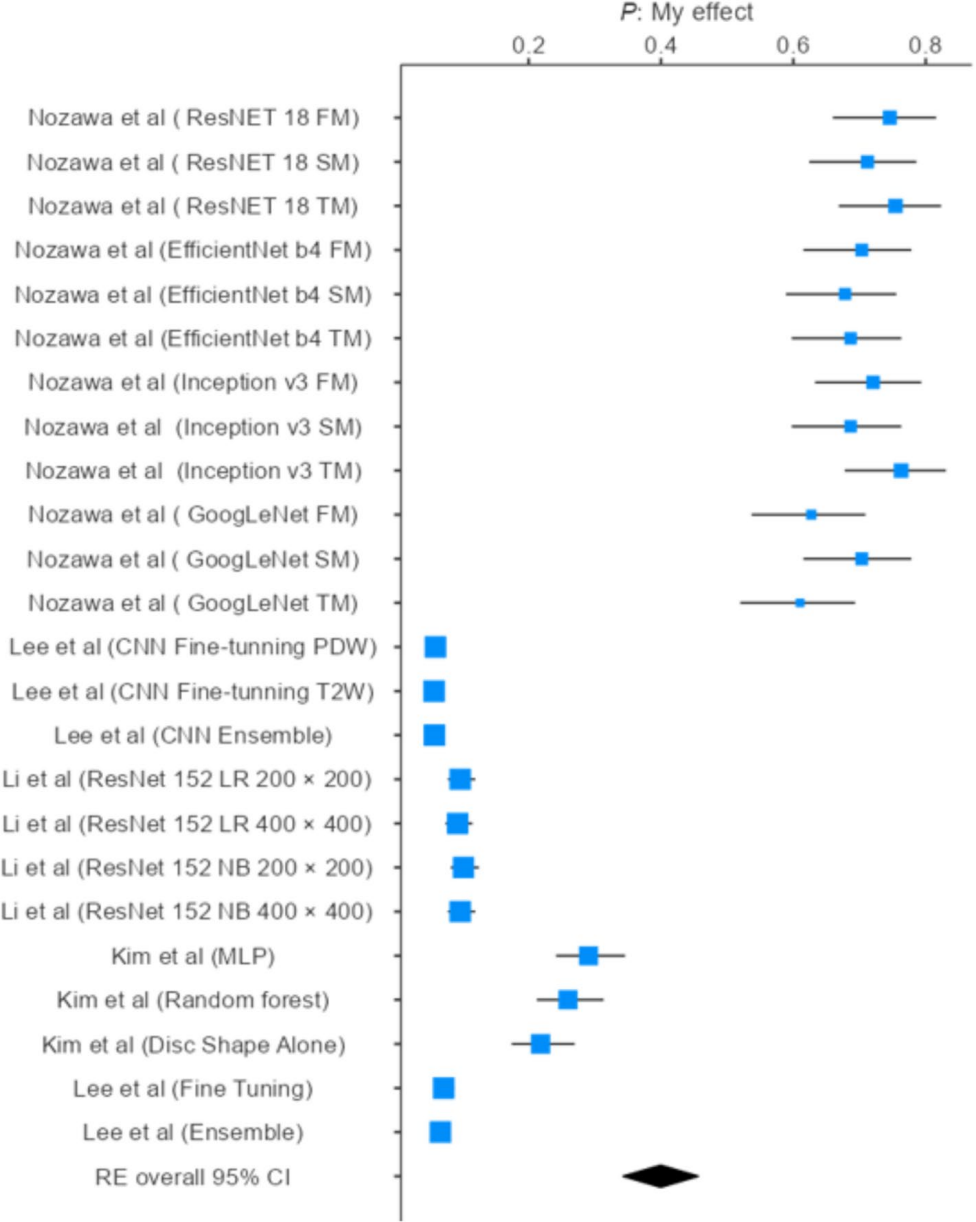
Specificity estimates of AI-based diagnostic models

A meta-analysis was performed on specificity data from 24 datasets, from 5 studies including various deep learning and machine learning models such as ResNet 18, EfficientNet b4, Inception v3, GoogLeNet, MLP, Random Forest, and ensemble approaches. Individual study specificities ranged widely, from 0.0577 to 0.7627, reflecting substantial variation in model performance. Using a random-effects model, the pooled specificity was estimated at 0.399 (95% CI 0.343–0.456). Heterogeneity was considerable, with an I^2^ of 99.19%, indicating that the variability across studies is largely due to differences in model architecture, dataset characteristics, imaging modalities, and methodological approaches rather than chance. The forest plot illustrates this variability, highlighting clusters of higher specificity in certain ResNet, EfficientNet, and Inception models, whereas ensemble and fine-tuned CNN models reported consistently lower specificity. These findings underscore the importance of model selection and dataset characteristics in determining specificity outcomes [18–20, 23, 25] (Fig. 6).

### Subgroup analysis of the sensitivity-based models

#### Subgroup analysis of ResNet 18 model

A subgroup meta-analysis was conducted for the ResNet 18 model variants (FM, SM, and TM) to evaluate pooled specificity. Individual study specificities ranged from 0.712 to 0.754, reflecting relatively consistent performance across the different ResNet 18 configurations. Using a fixed-effect model, the pooled specificity was estimated at 0.738 (95% CI 0.693–0.783), indicating moderate-to-high specificity. Heterogeneity was low in this subgroup (Diamond Ratio = 1.00), supporting the appropriateness of the fixed-effect model. The forest plot demonstrates the close clustering of individual study estimates, suggesting that the ResNet 18 architecture yields reproducible specificity outcomes across different dataset configurations. These results highlight the reliability of ResNet 18 models in terms of specificity compared to other architectures with greater variability [23] (Supplementary Fig. 12).

#### Subgroup analysis of EfficientNet b4 model

A subgroup meta-analysis was performed for the EfficientNet b4 model variants (FM, SM, and TM) to assess pooled specificity. Individual study estimates ranged from 0.678 to 0.703, indicating consistent performance across different configurations. Using a fixed-effect model, the pooled specificity was calculated as 0.689 (95% CI 0.642–0.737), demonstrating moderate-to-high specificity. The low heterogeneity observed (Diamond Ratio = 1.00) supports the use of a fixed-effect model, suggesting that the EfficientNet b4 architecture provides stable and reproducible specificity outcomes. The forest plot shows that individual study results are closely clustered around the pooled estimate, reinforcing the robustness of EfficientNet b4 for specificity measurement in this dataset [23] (Supplementary Fig. 13).

#### Subgroup analysis of GoogLeNet model

A subgroup meta-analysis was conducted for the GoogLeNet model variants (FM, SM, and TM) to evaluate pooled specificity. Individual study estimates ranged from 0.610 to 0.703, reflecting some variability across model configurations. Using a fixed-effect model, the pooled specificity was calculated as 0.649 (95% CI 0.600–0.698). The heterogeneity was low to moderate (Diamond Ratio = 1.17), supporting the application of a fixed-effect model. The forest plot demonstrates that most study estimates cluster around the pooled effect size, indicating that GoogLeNet maintains a reasonably consistent specificity across its different configurations [23] (Supplementary Fig. 14).

#### Subgroup analysis of inception v3 model

A subgroup meta-analysis was performed for the Inception v3 model variants (FM, SM, and TM) to determine pooled specificity. Individual study estimates ranged from 0.686 to 0.763, showing minor variation across configurations. Using a fixed-effect model, the pooled specificity was 0.725 (95% CI 0.679–0.771). The heterogeneity was low (Diamond Ratio = 1.00), indicating consistency across studies and supporting the use of a fixed-effect approach. The forest plot illustrates that the individual study estimates cluster closely around the pooled effect size, suggesting robust and consistent specificity for the Inception v3 model across its different configurations [23] (Supplementary Fig. 15).

#### Subgroup analysis of CNN models

A subgroup meta-analysis was conducted for the CNN fine-tuning models (PDW, T2W, and Ensemble). Individual specificity estimates were very low, ranging from 0.057 to 0.059, reflecting limited ability of these models to identify negative cases correctly. Using a fixed-effect model, the pooled specificity was 0.058 (95% CI 0.051–0.065). The heterogeneity was negligible (Diamond Ratio = 1.00), indicating consistent results across the three studies. The forest plot demonstrates tight clustering of the study estimates around the pooled effect, confirming low but consistent specificity for CNN fine-tuning approaches in this dataset [18] (Supplementary Fig. 16).

#### Subgroup analysis of ResNet 152 model

The subgroup meta-analysis of ResNet 152 models (LR and NB, 200 × 200 and 400 × 400 input sizes) revealed consistently low specificity values across the studies, ranging from 0.093 to 0.102, indicating limited performance in correctly identifying negative cases. Using a fixed-effect model, the pooled specificity was 0.097 (95% CI 0.086–0.107). Heterogeneity was minimal (Diamond Ratio = 1.00), suggesting homogeneity in the specificity estimates across the four studies. The forest plot illustrates a tight clustering of the individual study estimates around the pooled effect size, confirming low but consistent specificity for ResNet 152 models in this dataset [25] (Supplementary Fig. 17).

## Discussion

This systematic review and meta-analysis assessed the diagnostic performance of artificial intelligence (AI) models utilizing MRI for the detection of TMJ anomalies. Models reporting overall diagnostic outcomes were analyzed, focusing on core performance metrics, accuracy, sensitivity, and specificity without explicitly distinguishing between training, validation, and testing datasets. Pooled estimates were derived to evaluate the aggregate diagnostic performance of AI-based approaches. Furthermore, subgroup analyses were undertaken to investigate heterogeneity among different AI architectures and study populations, revealing substantial variations in model performance.

### Diagnostic accuracy

A total of 18 AI models from four included studies were evaluated for diagnostic accuracy in detecting TMJ anomalies using MRI. Individual model performance varied substantially, ranging from 0.051 (95% CI 0.041–0.064; Lee et al., Fine-Tuning) to 0.746 (95% CI 0.660–0.816; Nozawa et al., ResNet-18 FM). This wide range reflects considerable variability in model effectiveness [20, 23, 25]. Models based on ResNet and Inception architectures consistently demonstrated higher accuracies (0.69–0.75), suggesting that deep convolutional structures are particularly effective in capturing relevant imaging features of TMJ anomalies. In contrast, ensemble and fine-tuning approaches generally exhibited lower accuracy (< 0.10), possibly due to overfitting or limited generalization across datasets [20, 23, 25].

The pooled estimate of diagnostic accuracy across all models (*k* = 18), calculated using a random-effects model, was 0.487 (95% CI 0.403–0.571), indicating moderate overall performance of AI-based methods. Substantial heterogeneity was observed (*I*^2^ = 99.36%; *τ*^2^ = 0.0519; *H*^2^ = 257.9), underscoring the influence of architectural design, dataset variability, and training strategies on diagnostic performance. This high degree of heterogeneity suggests that pooled estimates should be interpreted with caution, as they may not accurately reflect the performance of individual model classes [20, 23, 25].

### Subgroup analysis of accuracy by model architecture

Subgroup analyses revealed that diagnostic accuracy varied markedly by model architecture. ResNet-18 models achieved the highest pooled accuracy across three datasets (0.732; 95% CI 0.686–0.778) with negligible heterogeneity (*I*^2^ = 0%), demonstrating robust and reproducible performance. This finding highlights the suitability of ResNet-18 for TMJ anomaly classification and its potential clinical applicability [23].

Similarly, Inception v3 models yielded a pooled accuracy of 0.712 (95% CI 0.666–0.759), and EfficientNet b4 models achieved 0.701 (95% CI 0.654–0.748), both with minimal heterogeneity, reflecting stable predictive capability. GoogLeNet models also showed consistent results (pooled accuracy 0.669; 95% CI 0.621–0.718), although slightly lower than ResNet-18 and Inception v3 [23]. In contrast, ResNet-152 models demonstrated poor performance (pooled accuracy 0.0862; 95% CI 0.0763–0.0961) despite variations in input resolution and learning configurations, indicating limited suitability for TMJ anomaly detection [25].

Collectively, these findings suggest that moderately deep architectures such as ResNet-18 and Inception v3 provide superior and consistent diagnostic performance, while overly complex or inadequately optimized networks (e.g., ResNet-152) may underperform [23, 25]. Standardizing image preprocessing, training, and validation protocols is therefore essential to enhance reproducibility and ensure clinically meaningful deployment of AI-based diagnostic tools in TMJ imaging [29–31]**.**

### Diagnostic sensitivity

A total of 29 AI models from six studies were included in the meta-analysis to evaluate the sensitivity of detecting TMJ anomalies. Individual model sensitivities varied widely, ranging from 0.035 to 0.746, reflecting differences in model architecture, training strategies, and dataset characteristics. The pooled sensitivity across all models was 0.399 (95% CI 0.348–0.450), with substantial heterogeneity (*I*^2^ = 99.23%), indicating pronounced variability in predictive performance across studies [17–20, 23, 25].

### Subgroup analysis of sensitivity

Subgroup analyses revealed that sensitivity was highly dependent on model architecture. Models based on the U-net family (U-net, U-net +  + , and U-net 3 +) demonstrated moderate pooled sensitivity (0.401; 95% CI 0.353–0.450). Although heterogeneity was moderate (*I*^2^ = 71.6%), this suggests that optimizing training parameters and preprocessing techniques could further improve their performance. In contrast, DeepLabV3 + and SegNet models exhibited lower pooled sensitivities (0.326; 95% CI 0.283–0.369), indicating limited capability to capture subtle imaging features associated with TMJ pathology [17].

More advanced architectures, including GoogLeNet, EfficientNet b4, and Inception v3, consistently achieved moderate-to-high sensitivities (0.689, 0.714, and 0.697, respectively). The relatively low-to-moderate heterogeneity observed within these subgroups indicates robust reproducibility and suggests that deeper models incorporating residual connections and multi-scale feature extraction offer significant advantages for clinical application. Among all evaluated models, ResNet-18 achieved the highest pooled sensitivity (0.724; 95% CI 0.677–0.770) with negligible heterogeneity, underscoring its robustness and potential as a reliable tool for early TMJ anomaly detection [23].

Conversely, simpler or less optimized approaches, including MLP, Random Forest, Disc Shape Alone, Fine-Tuning, Ensemble methods, and ResNet-152, demonstrated consistently low pooled sensitivities (ranging from 0.0375 to 0.300). Although heterogeneity was minimal, the uniformly poor performance of these models highlights their limited clinical utility, as low sensitivity increases the likelihood of false negatives and missed diagnoses [18–20, 25].

Overall, these findings indicate that diagnostic sensitivity is strongly influenced by model architecture. Advanced deep learning models such as ResNet-18, EfficientNet b4, and Inception v3 outperform simpler or fine-tuned networks, demonstrating superior capability in detecting subtle TMJ anomalies [23]. The high sensitivity achieved by these models suggests potential clinical value in supporting early diagnosis and reducing under-detection. These results emphasize the importance of selecting appropriate architectures, optimizing training pipelines, and implementing standardized validation protocols to ensure reliable and reproducible AI-assisted TMJ diagnostics [29–31].

### Diagnostic specificity

A total of 24 datasets were included in the meta-analysis of specificity, encompassing a range of deep and machine learning architectures such as ResNet-18, EfficientNet b4, Inception v3, GoogLeNet, CNN fine-tuning, ResNet-152, Random Forest, and ensemble approaches. Individual study specificities varied widely, from 0.0577 to 0.7627, reflecting substantial differences in model capability to correctly identify negative cases.

Using a random-effects model, the pooled specificity across all models was 0.399 (95% CI 0.343–0.456). Heterogeneity was high (*I*^2^ = 99.19%), indicating that the observed variability was primarily attributable to differences in model architectures, datasets, and methodological designs rather than chance. These findings underscore the strong influence of model selection and dataset characteristics on diagnostic specificity [18–20, 23, 25].

### Subgroup analysis of specificity

Subgroup analyses revealed distinct performance differences across architectures. ResNet-18 achieved the highest and most consistent specificity, ranging from 0.712 to 0.754, with a pooled estimate of 0.738 (95% CI 0.693–0.783) and negligible heterogeneity, confirming its robustness across datasets. Comparable results were observed with Inception v3, which demonstrated a pooled specificity of 0.725 (95% CI 0.679–0.771) and closely clustered study estimates, indicating stable and reliable performance. EfficientNet b4 models also achieved moderate-to-high pooled specificity (0.689; 95% CI 0.642–0.737) with low heterogeneity, while GoogLeNet yielded slightly lower pooled specificity (0.649; 95% CI 0.600–0.698) and moderate heterogeneity, suggesting some variability between datasets [23].

In contrast, CNN fine-tuning models performed poorly, with pooled specificity as low as 0.058 (95% CI 0.051–0.065), while ResNet-152 exhibited similarly low values (pooled specificity 0.097; 95% CI 0.086–0.107). These results highlight that while advanced architectures such as ResNet-18, Inception v3, and EfficientNet b4 offer reliable and reproducible specificity, fine-tuned CNN and ResNet-152 models demonstrate limited ability to correctly identify negative cases, restricting their clinical applicability [20, 23, 25].

Overall, the specificity analysis indicates that advanced architectures, particularly ResNet-18, Inception v3, and EfficientNet b4, consistently achieved moderate-to-high specificity (0.689–0.738) with low heterogeneity, reflecting their strong ability to accurately identify non-pathological cases and reduce false-positive diagnoses in TMJ imaging. GoogLeNet also performed reasonably well, though with slightly greater variability, suggesting moderate reliability [23]. Conversely, models such as ResNet-152 and CNN fine-tuning exhibited very low specificity, often below 0.10, posing a risk of overdiagnosis and unnecessary follow-up investigations [20, 25]. Clinically, high specificity is essential to avoid misclassification of healthy individuals, which can lead to anxiety, overtreatment, and increased healthcare costs. These findings suggest that only select advanced architectures currently demonstrate the reliability required for clinical translation. Further optimization of training strategies, along with access to diverse and high-quality datasets, is necessary to enhance specificity and ensure robust, reproducible diagnostic performance across populations [29–31].

Several recent reviews have explored the role of artificial intelligence in temporomandibular joint diagnostics. Jha et al. [32], provided an early multimodal overview across CBCT, panoramic, MRI, and nonimage datasets but limited quantitative synthesis to accuracy. Xu et al. [33] and Farook and Dudley [34] focused primarily on radiographic and multimodal imaging, respectively, reporting high diagnostic performance for osseous changes but limited MRI representation. Sankar et al. [35] specifically reviewed MRI-based AI models for disc displacement, though the analysis remained descriptive without pooled estimates. Mehta et al. [36], later summarised these findings through an umbrella review, emphasising the need for modality-specific quantitative evaluation.

The present study addresses this gap by providing the first MRI-exclusive meta-analysis quantifying pooled accuracy, sensitivity, and specificity across 18–29 AI models. Through subgroup and heterogeneity analyses, it demonstrates performance variability among architectures such as ResNet-18, Inception v3, and EfficientNet-b4. This focused, quantitative approach refines prior qualitative evidence, offering clinically relevant insights into the diagnostic reliability and translational potential of AI in TMJ imaging.

The findings of this review have clear clinical implications. By delineating AI architectures that demonstrate superior and more consistent diagnostic performance, particularly ResNet-18, Inception v3, and EfficientNet-b4, this study provides clinicians with robust, evidence-based insight into the models most suited for reliable application in TMJ MRI interpretation. The pooled estimates of sensitivity and specificity further assist clinicians in critically appraising AI-derived outputs, recognizing both their diagnostic potential and current methodological limitations. Collectively, these observations support the judicious and evidence-informed integration of AI-assisted tools into clinical TMJ diagnostic pathways.

## Limitations

Several limitations should be considered when interpreting these findings. The included studies exhibited substantial heterogeneity in dataset size, imaging protocols, preprocessing methods, and reporting of training, validation, and testing phases, which limits the generalizability of the pooled estimates. Most studies employed retrospective designs and lacked external validation, thereby increasing the risk of selection and optimism bias. The limited number of datasets for certain model architectures and inconsistent reporting of diagnostic performance metrics further constrain the strength of the conclusions, while selective reporting of only high-performing models cannot be excluded. Additionally, multiple models derived from the same study may have introduced statistical dependence that was not fully accounted for in the pooled analysis. Variability in MRI acquisition parameters, absence of standardized preprocessing pipelines, and lack of explainability metrics also reduce reproducibility and hinder model interpretability. Moreover, the inclusion of only English-language studies may introduce language bias, and the overall lack of prospective, multicenter validation limits the direct clinical applicability of these findings. The small number of included studies reflects the stringent eligibility criteria applied to ensure diagnostic validity and reproducibility. Most excluded records did not meet these methodological standards, such as the absence of independent testing or the use of non-MRI data.

## Future recommendations

To address these gaps, future research should prioritize prospective, multicenter studies using standardized MRI acquisition protocols and harmonized preprocessing pipelines to minimize methodological variability. Rigorous external validation across diverse populations, coupled with transparent reporting of all model development stages and performance metrics, is essential to enhance reproducibility and enable meaningful comparison between AI architectures. Incorporating explainable and interpretable AI approaches will further support clinical trust and integration into diagnostic workflows. In addition, open-access data repositories and adherence to established reporting guidelines, such as TRIPOD-AI and CLAIM, will promote transparency and facilitate benchmarking of models. Collectively, these measures will enable the development of robust, generalizable, and clinically applicable AI models for TMJ anomaly detection on MRI.

## Conclusion

The, advanced deep learning architectures such as ResNet-18, EfficientNet-b4, and Inception-v3 demonstrated comparatively higher diagnostic performance for detecting temporomandibular joint anomalies on MRI, achieving moderate-to-high pooled accuracy, sensitivity, and specificity. These findings suggest that AI-assisted image interpretation could support radiologists by improving diagnostic consistency, reducing reporting time, and aiding early detection of TMJ pathology. However, substantial heterogeneity, retrospective study designs, and limited external validation restrict current clinical applicability. To enable safe translation into practice, future research should prioritise prospective multicenter studies using standardized MRI protocols, transparent model reporting, and rigorous external validation. Clinically, integrating reliable AI tools within TMJ diagnostic workflows could enhance efficiency, reproducibility, and patient care outcomes in maxillofacial radiology.

## Data Availability

All data generated or analyzed during this study are included in this published article and its supplementary information files.

## Abbreviations

AI: Artificial intelligence
MRI: Magnetic resonance imaging
TMJ: Temporomandibular joint
ML: Machine learning
DL: Deep learning
AUC: Area under the curve
PPV: Positive predictive value
QUADAS: Quality assessment of diagnostic accuracy studies
CI: Confidence interval
I^2^: Inconsistency index (measure of heterogeneity)
τ^2^: Between-study variance (tau-squared)
SE: Standard error
SD: Standard deviation
PRISMA: Preferred reporting items for systematic reviews and meta-analyses
PROSPERO: International prospective register of systematic reviews
FM: Full model
SM: Simplified model
TM: Transfer model
LR: Learning rate
NB: Naive bayes
DC: Dice coefficient
HD: Hausdorff distance
TR: Repetition time
TE: Echo time
NEX: Number of excitations
PD: Proton density
CNN: Convolutional Neural Network
MLP: Multilayer perceptron
YOLO: You Only Look Once (object detection model)
CLAIM: Checklist for Artificial Intelligence in Medical Imaging
TRIPOD-AI: Transparent reporting of a multivariable prediction model for individual prognosis or diagnosis artificial intelligence extension
